# Exploring Anti-Coincidence Technique for Scattering Contamination Rejection in a Simultaneous PET/SPECT Imaging System

**DOI:** 10.1109/trpms.2025.3649623

**Published:** 2025-12-30

**Authors:** Yifei Jin, Junwei Du, Simon R. Cherry, Ling-Jian Meng

**Affiliations:** Department of Nuclear, Plasma and Radiological Engineering, University of Illinois at Urbana-Champaign, Urbana, IL 61801 USA; Department of Biomedical Engineering, University of California at Davis, Davis, CA 95616 USA; Department of Biomedical Engineering, University of California at Davis, Davis, CA 95616 USA; Department of Nuclear, Plasma and Radiological Engineering, the Department of Bioengineering and Beckman Institute for Advanced Science and Technology, University of Illinois at Urbana-Champaign, Urbana, IL 61801 USA

**Keywords:** Coincidence detection, down-scatter correction, PET, SPECT

## Abstract

Simultaneous PET/SPECT imaging instruments could enable comprehensive molecular characterization by combining the very high sensitivity and spatial resolution of PET with the ability to image a broad range of radionuclides, including therapeutic isotopes with SPECT. While PET and SPECT hardware can be integrated into a single hardware system, one of the major challenges in simultaneous PET/SPECT imaging is the down-scatter contamination from PET tracers, which can significantly degrade SPECT image quality and quantitative accuracy. In this study, we evaluated the efficacy of using anti-coincidence and active shielding techniques to reject the down-scatter contamination in SPECT data. Using GEANT4 simulations, we analyzed the performance tradeoffs resulting from different choices of active shielding component design, coincidence time windows, and SPECT detector materials (CZT versus GAGG). The anti-coincidence technique effectively rejected contamination events, achieving a signal-to-noise (SNR) enhancement from 0.29 to 1.48 and noise equivalent count rate (NECR) improvement from 41.3 to 89.1 cps with moderate active shielding. Even without active shielding, anti-coincidence alone significantly improved SPECT image quality (SNR: 1.38; NECR: 87.0 cps), making it practical for systems where shielding is constrained. In reconstructed images, anti-coincidence improved contrast from 0.78 (raw) to 0.96 and reduced noise from 1508 to 257 counts, outperforming traditional triple-energy-window (TEW) correction (contrast: 0.84; noise: 855). The anti-coincidence technique is also compatible with post-processing scatter corrections, offering a promising strategy for future hybrid PET/SPECT systems in both preclinical and clinical settings.

## I. INTRODUCTION

Simultaneous pet/spect imaging is an emerging multimodality approach that combines PET’s high-sensitivity metabolic imaging with SPECT’s ability to image a broad range of radiotracers, including therapeutic radionuclides [1], [2]. It could offer a comprehensive platform for multifunctional molecular imaging [3], [4], [5], [6]. For instance, in Parkinson’s disease, this combined approach more accurately differentiates between Parkinsonian syndromes by simultaneously mapping cerebral glucose metabolism (F-18-FDG PET) and dopamine transporter activity (I-123 SPECT), providing a more comprehensive assessment than either modality alone [7], [8], [9]. This multimodality is also attractive for the rapidly evolving field of personalized theragnostic—for example, in advanced prostate cancer treated with radioligands like Lu-177 and Ac-225 [10], [11], [12], [13]. These therapeutic isotopes emit gamma photons that can be imaged with SPECT, while PET tracers can concurrently assess metabolism or receptor expression. This supports real-time, patient-specific dosimetry and treatment planning, which is critical in the shift toward personalized theragnostic. A simultaneous PET/SPECT system could further enhance theragnostic applications by allowing a PET tracer (e.g., FDG or a receptor-specific PET agent) to be imaged at the same time as a therapeutic radiotracer—giving real-time correlation between tumor metabolism and therapy uptake. In thyroid oncology, for example, simultaneous imaging of I-131/I-123 SPECT and F-18-FDG PET has been shown to differentiate tumor grades: high-grade tumors often have high FDG and low I-123/I-131 uptake, whereas low-grade tumors show the opposite [14], [15].

One of the major challenges in simultaneous PET/SPECT systems is down-scatter contamination from PET tracers, which could significantly degrade the SPECT image quality and compromise its quantitative accuracy. Many scatter correction methods have been explored over the years to address this down-scatter issue. Energy-window-based methods, such as the triple-energy-window (TEW) method, estimate scatter contamination using auxiliary energy windows [16], [17], [18], [19], [20], [21]. While simple to implement, these approaches may perform poorly in low-count scenarios, and depend critically on scaling factors influenced by patient anatomy and detector properties [22]. Spectrum-based methods leverage the full energy spectrum for scatter modeling, offering improved accuracy but remaining sensitive to statistical noise and requiring precise spectral calibration [23], [24], [25].

Advances in computational resources have facilitated the use of simulation-based correction methods, which predict scatter distributions via physics modeling and iterative reconstruction [22], [26], [27], [28], [29], [30], [31], [32], [33], [34], [35], [36], [37]. Though highly accurate, these methods demand significant computational resources and rely on prior anatomical information, typically from CT. More recently, deep learning approaches have emerged as a novel way to handle scatter and crosstalk correction [38], [39], [40], [41], [42]. Compared to simulation-based methods, once a network is trained, it can perform scatter correction extremely fast. However, the limitations include generalization and robustness, and the requirement of training data [43].

A common limitation across these methods is their dependence on high-count statistics, rendering them ineffective in low-activity scenarios, such as SPECT imaging with Ac-225 [44], [45], [46]. A few recent studies have explored coincidence-based methods for reducing crosstalk in multi-isotope imaging, such as the Compton–PET hybrid systems [47], [48]. Our group also introduced a coincidence detection framework to intrinsically reject down-scattered and crosstalk events in low-activity cases [49]. While effective for tracers emitting cascade photons (e.g., Lu-177 and Ac-225), this method is restricted to specific radionuclides.

In this work, we propose an anti-coincidence (abbreviated as anti-C in figures) technique combined with active shielding to fundamentally reduce down-scatter contamination in SPECT imaging. By detecting coincidence events, the method identifies and rejects scattered photons during acquisition. Active shielding enhances the detection efficiency of coincidences, ensuring robust performance across diverse tracers. To evaluate this approach, we propose an integrated PET/SPECT system design in which SPECT detectors are embedded within the gaps between PET detector blocks, enabling simultaneous data acquisition. This article is organized as follows: *First*, we present the theoretical framework for anti-coincidence PET/SPECT system design. *Second*, we develop a simulation study to evaluate the proposed scatter correction technique. *Third*, we evaluate the impact of the timing resolution, coincidence time window, and system count rate on the efficacy of the active shielding. *Finally*, we extend the Monte Carlo study to compare the use of different detector materials (CZT versus GAGG) for the SPECT subsystem.

## II. Material and Methods

### A. Proposed Simultaneous PET/SPECT System Design With Micro-Gamma-Cameras Embedded in a Long-FOV PET Scanner

**Fig. 1.**
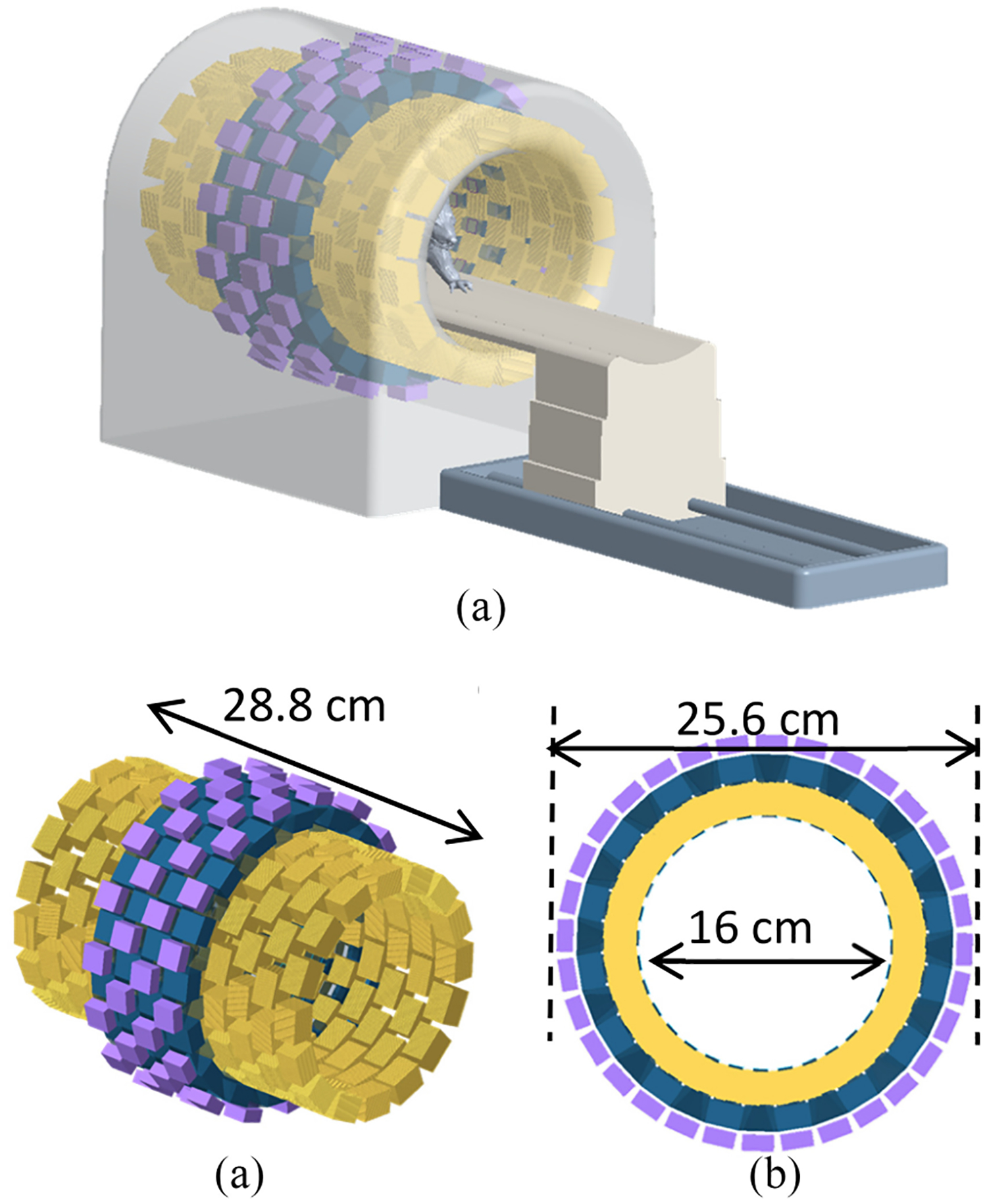
Schematic of the proposed preclinical PET/SPECT system. (a) Overall system design showing PET detectors (yellow) and SPECT cameras (purple). (b) Perspective view, illustrating the axial coverage of 28.8 cm. (c) Transverse view showing the inner and outer diameters (16 and 25.6 cm, respectively).

In this study, we propose a simultaneous PET/SPECT system design for preclinical applications as shown in Fig. 1. The system has a total length of 28.8 cm with a center-to-center spacing of 2.4 cm between adjacent rings. The inner and outer diameters are 16 and 25.6 cm. It comprises 12 rings, including 8 full PET rings (4 on each end). Each ring contains 16 BGO detectors measuring 20 mm (axial) × 28 mm (tangential) × 20 mm (radial). Each PET detector module consists of a 20 × 28 BGO array with an 1.0-mm pitch and 20-mm thickness. To improve angular sampling, adjacent rings are offset by 11.25°. The full rings in Fig. 1(c) result from the superimposition of multiple angular offset detector rings, which collectively provide complete angular sampling despite individual ring gaps.

The 4 central rings are hybrid PET/SPECT rings. Each with 16 BGO detector blocks of 20 mm (axial) × 20 mm (tangential) × 20 mm (radial) in size (20 × 20 BGO array). Compared to the BGO detectors in full PET rings, this shorter tangential length leaves gaps between adjacent PET detectors in the 4 central rings.

The SPECT subsystem has 64 CZT pinhole cameras to fit the gap. Each SPECT component utilizes 1-mm diameter loft hole collimators paired with 1-cm-thick CZT detectors. These detectors measure 2.2 × 2.2 × 1 cm^3^ and offer a very high sensitivity and approximately 0.5-mm FWHM spatial resolution in 3 dimensions, making them highly effective for detailed SPECT imaging. The SPECT subsystem employs a minification factor of 1:4 and offers ~0.05% sensitivity and 1~1.5 mm spatial resolution with a field-of-view (FOV) of 6 cm in diameter and 9-cm axial length. This PET subsystem can achieve ~14% sensitivity at the center of the scanner and ~1-mm spatial resolution across the FOV of 8 cm in diameter and 28 cm. Compared to a PET configuration without SPECT inserts, the small inter-ring gaps in the central 4 rings slightly reduce the center sensitivity from 16.8% to 14%, as the long axial length compensates for this loss. The insertion of SPECT detectors does not affect the intrinsic PET spatial resolution, which is primarily determined by the crystal size and the detector ring diameter.

### B. Scattering-Rejection With Anti-Coincidence Technique and Active Shielding

The principle of the anti-coincidence technique is to identify and reject contamination events that mimic true SPECT signals. In this context, we define *gamma rays of interest* as those photons emitted by SPECT tracers that are intended for imaging. In SPECT imaging, a predefined energy window is used to select *true events* (➁ in Fig. 3) originating from gamma rays of interest without prior scattering. However, in simultaneous PET/SPECT imaging—or in other scenarios involving multiple gamma-ray energies—higher-energy photons, such as 511-keV annihilation photons from PET tracers, may deposit only a fraction of their initial energy in the SPECT detector due to energy loss before reaching the detector (e.g., through interactions in the object, surrounding materials, or PET detectors) or due to partial energy deposition via Compton scattering within the SPECT detector itself. If this partial energy falls within the SPECT energy window, the event can be misclassified as a true SPECT signal, introducing *contamination events* (➂➃ in Fig. 3).

For a gamma ray of interest, it should reach the detector without significant energy loss. Any prior interaction, such as Compton scattering, would shift its energy outside the SPECT window, thus excluding it from imaging. In contrast, contamination events from higher-energy photons require energy loss—either through scattering elsewhere in the system or through partial energy deposition in the SPECT detector. These unwanted photons may interact in PET detectors or the active shielding detectors, producing *coincidences* with the SPECT detection (➂ in Fig. 3, various coincidence pathways are illustrated).

**Fig. 2.**
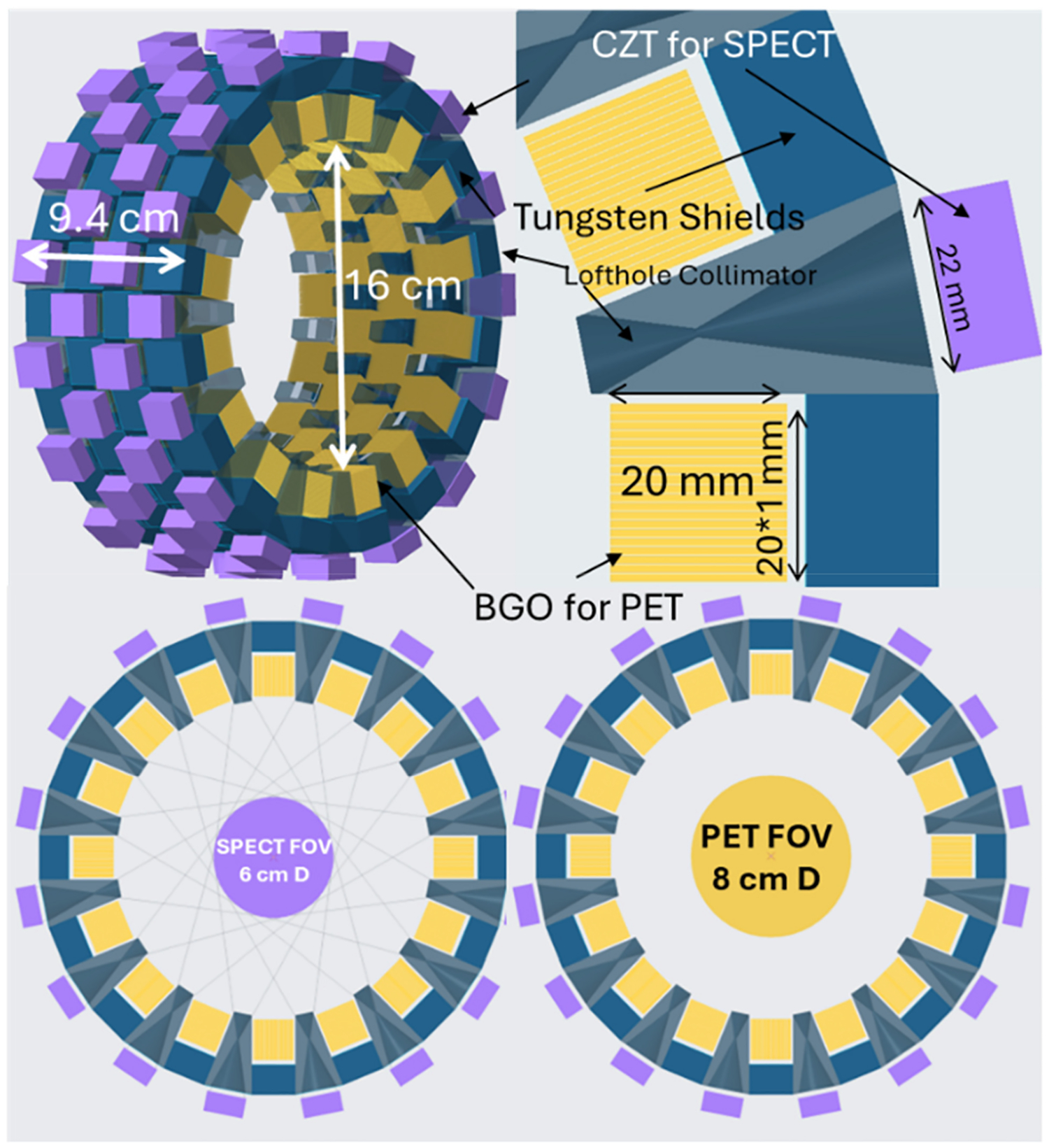
Detailed design of the proposed preclinical PET/SPECT system. A zoomed-in view shows the dimensions of PET modules (20 × 20 × 20 mm3) and SPECT modules (22 × 22 × 10 mm^3^) with tungsten lofthole collimators and shielding. The transverse FOV for SPECT is 6 cm in diameter, and for PET is 8 cm in diameter.

**Fig. 3.**
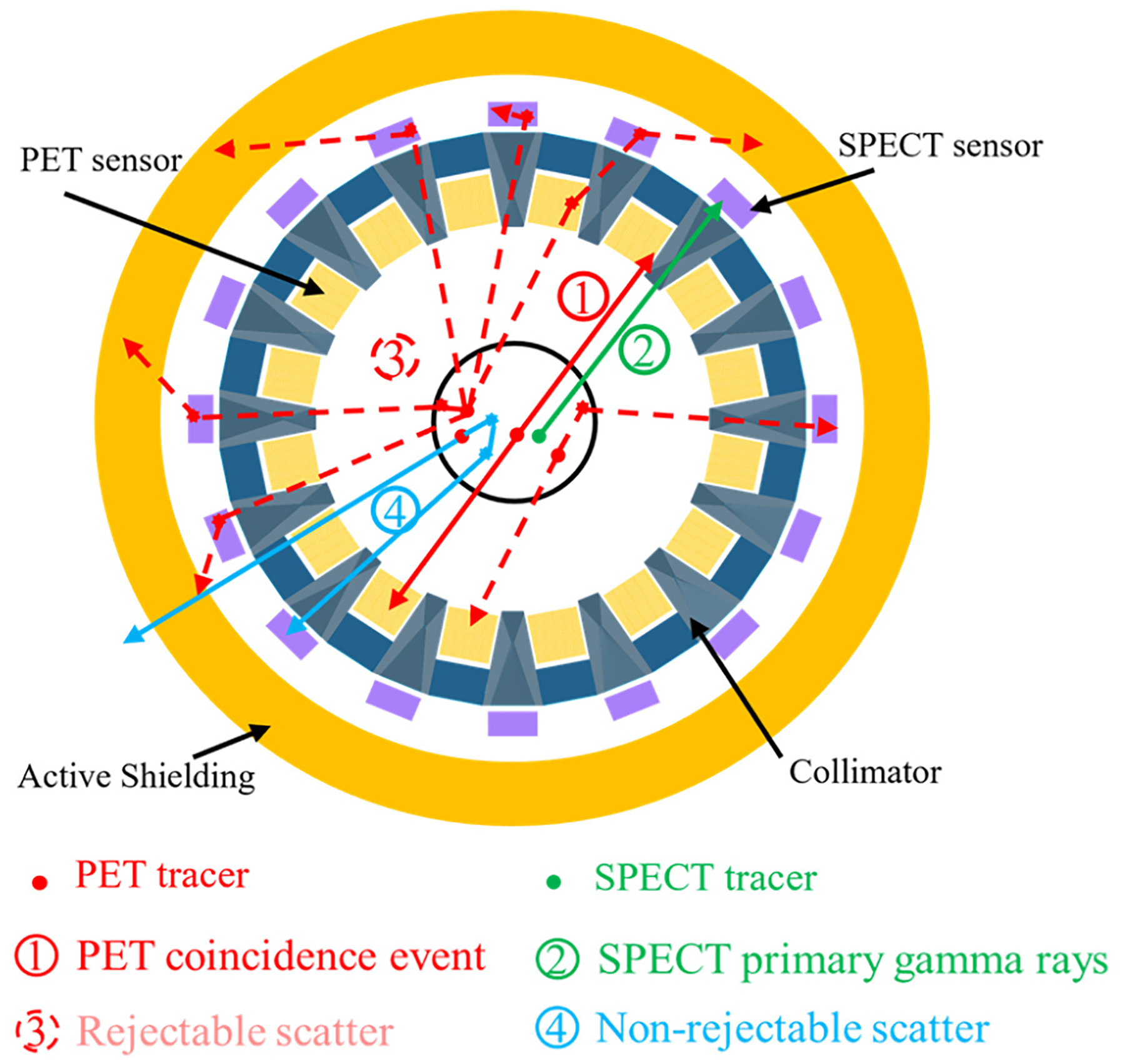
Schematic illustration of scatter rejection in the proposed PET/SPECT system. PET tracers (red) emit 511-keV annihilation photons, which may lead to PET coincidence events (➀) and rejectable scatter events in the SPECT energy window (➂, nondetected annihilation photons are omitted for simplicity). SPECT tracers (green) emit primary gamma rays (➁) that are detected without coincidence. Nonrejectable scatter events (➃, blue) represent cases where scattered photons mimic true SPECT signals without any detectable coincidence.

This distinction forms the foundation of the *anti-coincidence technique*: true events would *not* lead to any coincidence detection, except for random coincidence due to finite timing resolution, whereas contamination events are more likely to be accompanied by coincidence interactions. Such contamination events are therefore rejectable scatter events (➂ in Fig. 3), which can be rejected through anti-coincidence logic. To further reject contamination events, we explored the use of BGO as active shielding (nonposition-sensitive detectors) (Fig. 2) located around SPECT detectors to improve the chance of detecting interactions in coincidence with the contamination events, enabling their rejection through the anti-coincidence logic. Contamination events caused by 511-keV photons that scatter only in nonactive materials—such as objects and collimators—without generating detectable coincidences, are nonrejectable scatter events (➃ in Fig. 3).

Rejectable contamination events can be further classified into three types based on the detectors that register coincident interactions with the contamination events:

#### a) Coincidence With PET:

Annihilation photons, scattered or unscattered in the object, interact with PET detectors after scattering in PET detectors and/or collimators, then deposit full or partial energy in the SPECT primary energy window, with the scattered photon escaping the system. Alternatively, one annihilation photon scatters in nondetector materials and falls into the SPECT energy window, while its paired photon is detected by a PET detector.

#### b) Coincidence With SPECT:

Annihilation photons scatter in nondetector materials, then scatter within SPECT detectors, with either the primary or scattered photons depositing energy in the SPECT energy window. Alternatively, one annihilation photon scatters in nondetector materials and falls into the SPECT energy window, while its paired photon is detected by a SPECT detector.

#### c) Coincidence With Active Shielding:

Annihilation photons scatter in nondetector materials, then scatter within SPECT detectors and deposit partial energy in the SPECT energy window, with the scattered photon subsequently detected by active shielding. Alternatively, one annihilation photon scatters in nondetector materials and falls into the SPECT energy window, while its paired photon is detected by active shielding.

It is noted that, for a given contamination event, its coincident interactions may also be detected by multiple detectors (PET, SPECT, or active shielding) in coincidence.

### C. GEANT4 Simulation Setup

**Fig. 4.**
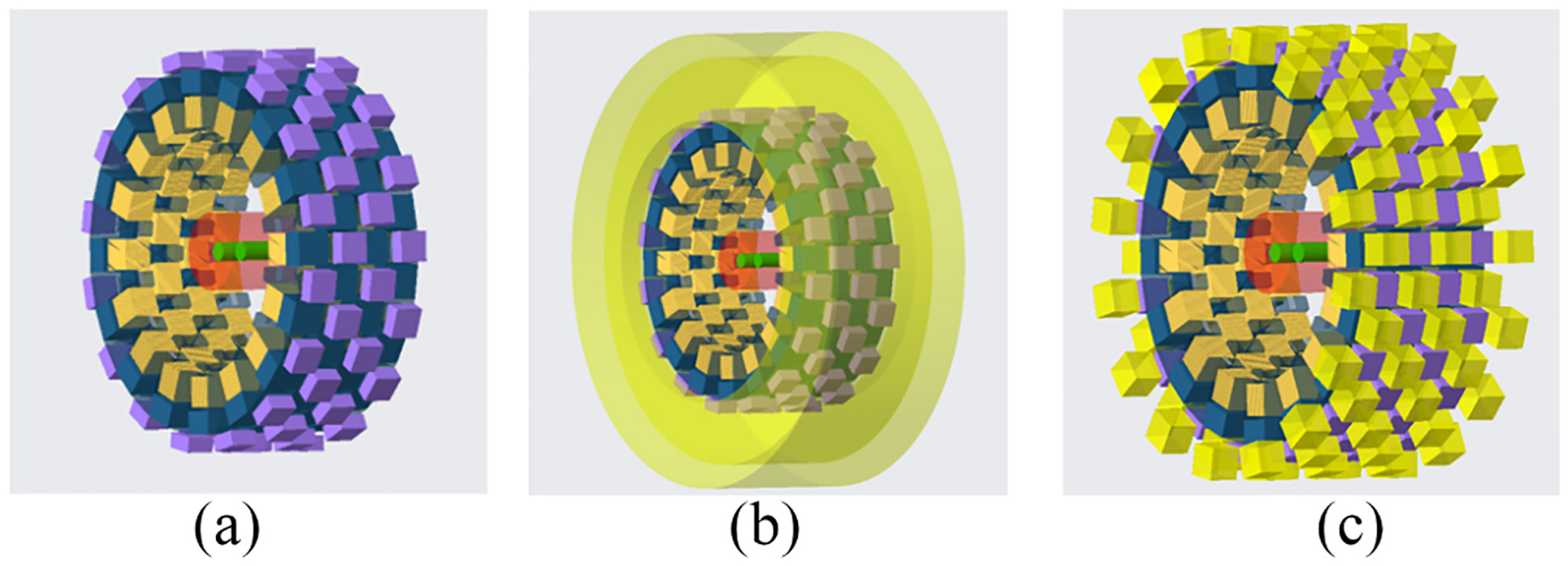
Three active shielding configurations evaluated in this study. (a) No active shielding. (b) Full active shielding covering the entire system. (c) Moderate active shielding.

In this study, we used GEANT4 simulation [50] to evaluate the anti-coincidence technique in three active shielding designs. First, Fig. 4(a) shows the basic system without additional active shielding. We used BGO detectors for PET and CZT detectors for SPECT, with their key parameters summarized in Table I. The BGO detectors employed 20% FWHM energy resolution and 1-ns FWHM timing resolution [51], while the CZT detectors used 2% FWHM energy resolution and 25-ns FWHM timing resolution [52], [53], [54]. A coincidence time window of [–30 ns, 30 ns] was applied for anti-coincidence logic. Second, we applied full active shielding to cover the whole system with 2-cm thick nonposition-sensitive BGO to maximize the chance to capture escaping scatter photons [Fig. 4(b)]. However, this design suffers from clear disadvantages, including high cost for shaping the BGO active shielding, difficulties in cooling and readout.

Finally, we evaluated a moderate active shielding design [Fig. 4(c)]. Each 10-mm thick DOI-capable CZT detector was virtually divided into two segments: the front 6 mm was used for SPECT imaging, while the remaining 4 mm at the back served as active shielding for anti-coincidence detection. BGO blocks were placed between the CZT detector modules to further enhance shielding. This configuration reduces costs while leaving space for cooling and readout electronics. In this study, we evaluated the efficacy of this approach against the use of the full-active shielding shown in Fig. 4(b).

**Fig. 5.**
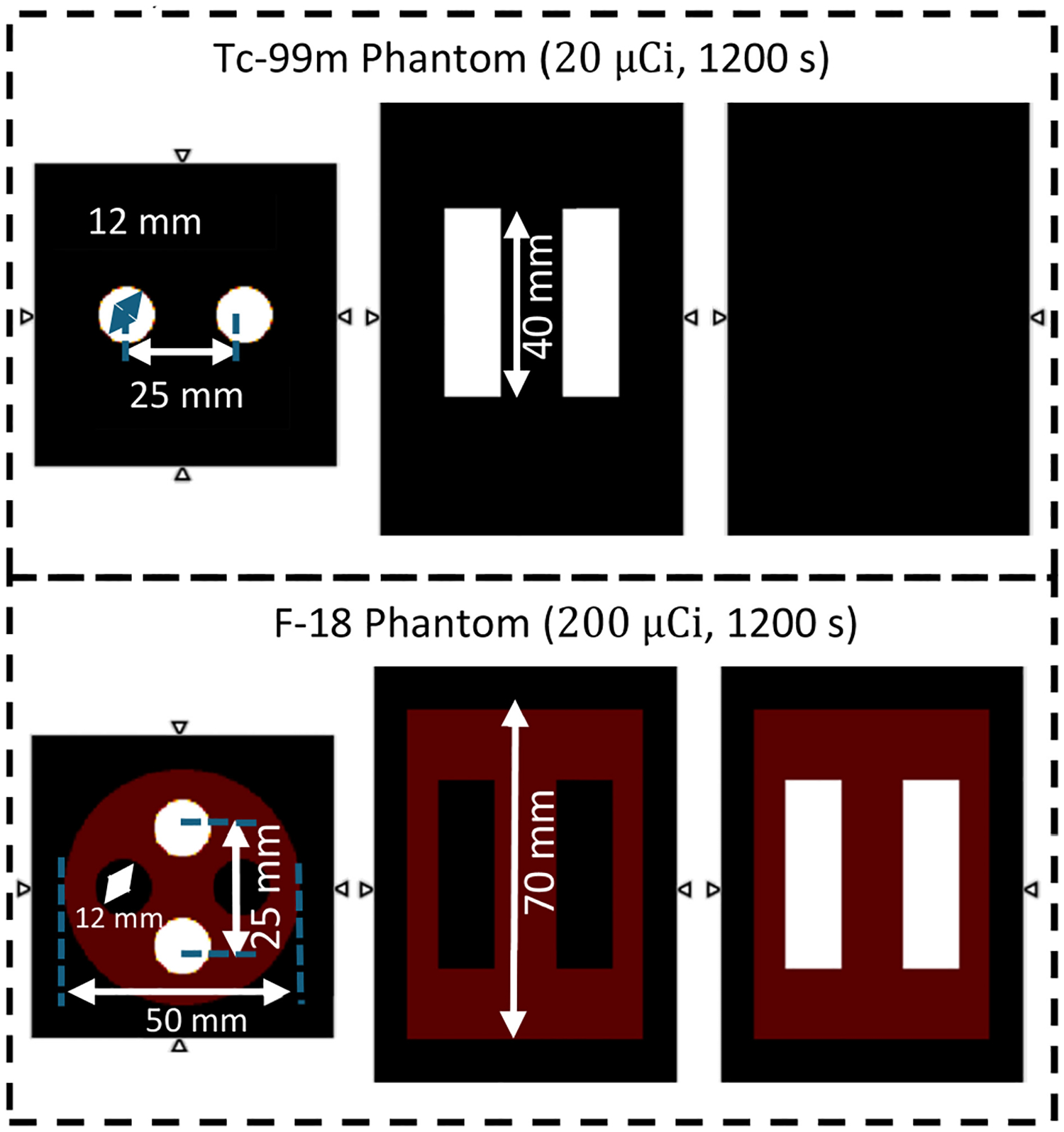
Dual-tracer phantom designs for simulation: Tc-99m (top) and F-18 (bottom) with hot rods and background regions.

We simulated an F-18/Tc-99 m dual-tracer phantom as shown in Fig. 5. It had two Tc-99m hot rods (diameter: 12 mm, separation: 25 mm) with a concentration of 4.4 μCi/mL and 20 μCi activity in total. The F-18 phantom contained 200 μCi activity in total. It had a continuous region of 1.36-μCi/mL concentration, 2 void rods and 2 hot rods (diameter: 12 mm, separation: 25 mm) with 5.46-μCi/mL concentration. A polycarbonate cylinder with 30-mm radius and 100-mm height was used as a scattering medium. The acquisition time was 20 min. This was to simulate limited uptake in some extreme cases. The SPECT energy window is set to [140 keV − 2*FWHM*, 140 keV + 2*FWHM*].

Note that in the GEANT4 simulation, event timestamps were not directly produced; instead, only event IDs were recorded. Timestamps were therefore generated in a manner consistent with the statistical properties of radioactive decay. For each radionuclide, the expected decay rate in each 1-s interval was determined by *λ* = *A* × *I*, where *A* is the source activity and *I* is the emission intensity (88.5% for 140-keV gamma rays from Tc-99m and 96.7% for positrons from F-18). The number of emitted photons/positrons in each second was then sampled from a Poisson distribution *N*_*s*_ *~* Poisson(λ), modeling the stochastic arrival of decay events. Note that time-of-flight (TOF) effects were not modeled because the SPECT detectors have insufficient timing resolution to support meaningful TOF discrimination. For each second *s*, the corresponding detection times for each event were drawn from a uniform distribution over [*s* − 1*, s*), so that the aggregated timestamp list preserves both the Poisson arrival statistics and the temporal ordering of events. A total of $\sum_{s}^{1200}N_{s}$ timestamps were created by MATLAB and associated with a unique event ID from GEANT4. Since not all simulated events resulted in measurable detections, only those corresponding to detected interactions were used in subsequent analysis.

To evaluate the anti-coincidence performance in a real scenario, a timing resolution blur was subsequently applied to each timestamp by adding a Gaussian blurring with FWHM equal to the detector timing resolution listed in Table I. To quantify the contributions of random and true coincidences, we generate time-difference spectra. All detected events were separated into: 1) primary events, defined as events detected by SPECT cameras and falling within the SPECT primary energy window and 2) secondary events, defined as all remaining events. Time-difference spectra were constructed by pairing each primary event with all secondary events occurring within a predefined time range (−100–100 ns) and accumulating the resulting Δ*t* = *t*_primary_ − *t*_secondary_ values. Because the tracer identity of each event is known, each primary → secondary pair was classified into one of four categories, such as Tc-99m → F-18, Tc-99m → Tc-99m, F-18 → Tc-99m, and F-18 → F-18. F-18 → F-18 category corresponds to true coincidence. The Tc-99m → F-18 and Tc-99m → Tc-99m categories represent random coincidences that contribute to *false rejection*, as primary events originating from Tc-99m are rejected by the anti-coincidence logic. The F-18 → Tc-99m category, although also arising from random coincidences, contributes to *true rejection* because the rejected primary events originate from F-18 contamination.

Performance was evaluated in the projection domain by quantifying the true rejection fraction and false rejection fraction, defined as the fractions of rejected contamination events and incorrectly rejected true SPECT events due to random coincidences, respectively. In addition, we evaluated the enhanced signal-to-noise ratio (SNR_enh_) and noise-equivalent count rate (NECR_enh_) as defined in Section II-D.

### D. Evaluation of the Impact of Coincidence Time Window

The coincidence time window has a direct impact on the efficacy of the proposed scattering rejection techniques. With a wider window, there is a higher chance of detecting coincidence events resulting in more rejection, which rejects more contamination events but meanwhile also leads to more true events rejected by random coincidence events. Hence, there is a tradeoff in selecting the coincidence time window. In this study, we used SNR = (signal*/*noise) and NECR = (signal^2^*/*signal + nois*e*) as metrics to evaluate the impact of the coincidence time window. Here, the *signal* and *noise* are defined as true SPECT counts and down-scattered contamination counts, respectively, in the projection domain. Because the true and scattered counts are known from simulation, this approach reflects intrinsic signal-to-noise characteristics independent of image reconstruction, making projection-domain SNR values inherently different from image-domain SNR values typically used to assess clinical image quality.

Consider a PET tracer with an activity of *Q* and a coincidence time window of [−*τ, τ*]. Other notations are as follows.

1. Coincidence time resolution: $\sum =\sqrt{\sigma_{\text{PET}}^{2}+\sigma_{\text{SPECT}}^{2}}$.
2. True count rate in SPECT primary window: *T*.
3. Probability of 511-keV annihilated photons scattered to SPECT primary window: *p*_1_.
4. Probability of scattered photons in SPECT primary window rejected by true anti-coincidence logic: *p*_2_*τ*, Σ).
5. Total count rate in SPECT primary window: *T + Qp*_1_.
6. Total count rate in anti-coincidence sensors (PET/SPECT out of primary window/Active shielding): *Qp*_2_.

*σ*_PET_ and *σ*_SPECT_ represent the FWHMs of the timing resolutions of PET and SPECT detectors, respectively. Assuming the time differences of coincidence events yield normal distribution *N*(0, (Σ*/*2.355)), *p*_2_(*τ*, Σ) can be computed as *k* erf $\left(2.355\tau /\sqrt{2}\sum \right)$, where *erf* is the error function, indicating *p*_2_ is positively correlated with *τ* and negatively correlated with Σ, and *k* ∈ [0, 1] is the intrinsic probability that the contamination event also produces a detectable coincidence event.

Before any enhancement, the raw signal and noise are signal_raw_ = *T*, noise_raw_ = *Qp*_1_, leading to SNR_raw_ = *(T/Qp*_1_) and NECR_raw_ = (*T*^2^*/T* + *Qp*_1_). With the anti-coincidence technique, the signal is

(1) $${\text{signal}}_{\text{enh}}=T\left(1-2Qp_{2}(\tau ,\Sigma )\tau \right)$$

due to random coincidence, and the noise becomes

(2) $${\text{noise}}_{\text{enh}}=Qp_{1}\left(1-p_{2}(\tau ,\Sigma )\right)\left(1-2Qp_{2}(\tau ,\Sigma )\tau \right).$$

Therefore, we can compute the SNR_enh_ and NECR_enh_ as

(3) $${\text{SNR}}_{\text{enh}}=\frac{T}{Qp_{1}\left(1-p_{2}(\tau ,\Sigma )\right)}$$

(4) $${\text{NECR}}_{\text{enh}}=\frac{T^{2}\left(1-2Qp_{1}\tau \right)}{T+Qp_{1}\left(1-p_{2}(\tau ,\Sigma )\right)}.$$

The enhancement of SNR can be expressed as

(5) $${\text{enh}}_{\text{SNR}}=\frac{{\text{SNR}}_{\text{enh}}}{{\text{SNR}}_{\text{raw}}}=\frac{1}{1-p_{2}(\tau ,\Sigma )}$$

while the enhancement of NECR can be expressed as

(6) $${\text{enh}}_{\text{NECR}}=\frac{{\text{NECR}}_{\text{enh}}}{{\text{NECR}}_{\text{raw}}}=\frac{\left(T+Qp_{1}\right)\left(1-2Qp_{1}\tau \right)}{T+Qp_{1}\left(1-p_{2}(\tau ,\Sigma )\right)}\propto \frac{\left(1-2Qp_{1}\tau \right)}{1+\frac{Q}{T}p_{1}\left(1-p_{2}(\tau ,\Sigma )\right)}.$$

According to (5), the enhancement of SNR is positively correlated with *τ* and negatively correlated with Σ, which means using a wider energy window or having better coincidence time resolution can always get better enhancement of SNR. The second line of (6) has been normalized by a factor (*T* + *Qp*_1_)*/T* to remove terms that are independent of *τ* and Σ since our goal is to evaluate the impact of coincidence time window and timing resolution. Given a fixed Σ, increasing *τ* can simultaneously: 1) increases the trueveto probability *p*_2_(*τ*, Σ), which reduces the contamination term in the denominator and 2) increases random-coincidence losses, which reduces the numerator. Hence, to achieve the best NECR, there is an optimal *τ* depending on the contamination burden relative to true counts, *Q/T*. Note that *p*_2_(*τ*, Σ) is monotonically increasing as *τ* increases (wider coincidence time window) and decreasing as Σ increases (worse timing). The optimal *τ* decreases with higher *Q/T* (requires a narrower window at higher PET rates) in order to reduce the rejection due to random coincidences and increases with better timing (smaller Σ).

In this study, we evaluated the optimal *τ* given three PET tracer activities: 50 μCi, 100 μ*Ci*, 200 μCi with the moderate active shielding design and fixed SPECT tracer activity of 20 μCi.

### E. Comparisons of Scatter Correction Methods and Detector Materials

#### 1) Anti-Coincidence Versus TEW Methods:

TEW scatter correction is a widely used traditional scatter correction method [55]. In this study, we compared the reconstructed images with the anti-coincidence technique in the *moderate* active shielding design and the TEW method in the *no* active shielding design. We chose no active shielding for the TEW method because additional active shielding might induce more down scatter to SPECT detectors, but the additional scatter contamination can be identified and rejected with the anti-coincidence technique. Note that the SPECT detectors have a total thickness of 10 mm and are equipped with DOI capability, allowing virtual segmentation of the thickness. We only used the first 6-mm thickness from the front surface for SPECT imaging, while the remaining 4 mm at the back was considered as active shielding. For consistency, we only used the first 6-mm thickness for SPECT imaging in the TEW method as well. We reconstructed images using the maximum-likelihood expectation-maximization (MLEM) algorithm [56].

#### 2) CZT Versus GAGG as SPECT Detector Materials:

One of the limitations of CZT detectors is the poor timing resolution (25 ns simulated in this study), which needs a wide coincidence time resolution and results in more false rejection through random coincidences. But it is still appealing because its high energy resolution allows for a narrower energy window to increase the SNR_raw_ and NECR_raw_. In contrast, with scintillation detectors like GAGG, although it can achieve a higher timing resolution allowing for a shorter coincidence time window and reducing the false rejection through random coincidences, its energy resolution is poor. To assess these tradeoffs, we implemented both detector types in GEANT4 simulations under the same system geometry (moderate active shielding design). The GAGG detectors were modeled with a timing resolution of 1 ns and an energy resolution of 10%. The coincidence time window [−2 *ns*, 2 *ns*] was selected to approximately have the best NECR. Performance was evaluated in both the projection and reconstruction domains. In the projection domain, we used the same criterion as mentioned in Section II-C.

We reconstructed images using identical pipelines and compared contrasts and noise levels. The contrast is defined as

(7) $$\text{Contrast}=\frac{\left|S_{1}-S_{2}\right|}{S_{1}}$$

where *S*_1_ and *S*_2_ are mean intensities of region-of-interest (ROI) 1 and ROI 2. ROI 1 was defined by shrinking the original hot rod regions in the Tc-99m phantom inward by 20% along their boundaries, retaining 80% of the original volume. Similarly, ROI 2 was defined by shrinking the active regions in the F-18 phantom inward by 20%. This operation excluded boundary voxels to reduce partial volume effects and emphasized the core signal *(S*_1_) and noise level *(S*_2_).

We also assessed how the detector properties influenced the effectiveness of anti-coincidence rejection compared to traditional TEW scatter correction.

## III. Results

### A. Data Qualities With the Anti-Coincidence Technique and Three Active Shielding Designs

**Fig. 6.**
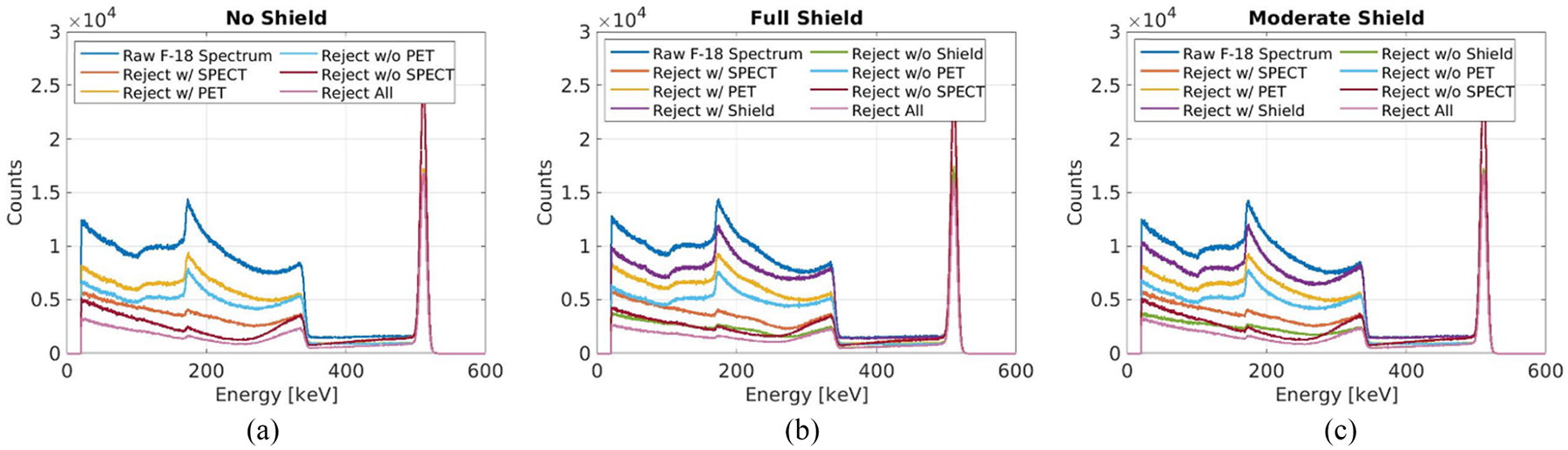
Energy spectra from F-18 acquired with SPECT detectors under different active shielding configurations. (a) No active shielding. (b) Full active shielding. (c) Moderate active shielding. For each configuration, spectra are shown for different combinations of detectors used in the anti-coincidence technique: no anti-coincidence (raw spectrum), PET only, SPECT only, active shielding only (when applicable), PET + SPECT, SPECT + active shielding (when applicable), PET + active shielding (when applicable), and all detectors combined.

To evaluate the effectiveness of the anti-coincidence technique under different shielding configurations, we first analyzed the energy spectra of F-18 acquired in the SPECT detectors (Fig. 6). The raw energy spectra show a substantial number of scattered events falling within the SPECT primary energy window around 140 keV. By applying anti-coincidence rejection logic with different combinations of coincidence detectors (SPECT, PET, and active shielding), contamination levels were progressively reduced.

**Fig. 8.**
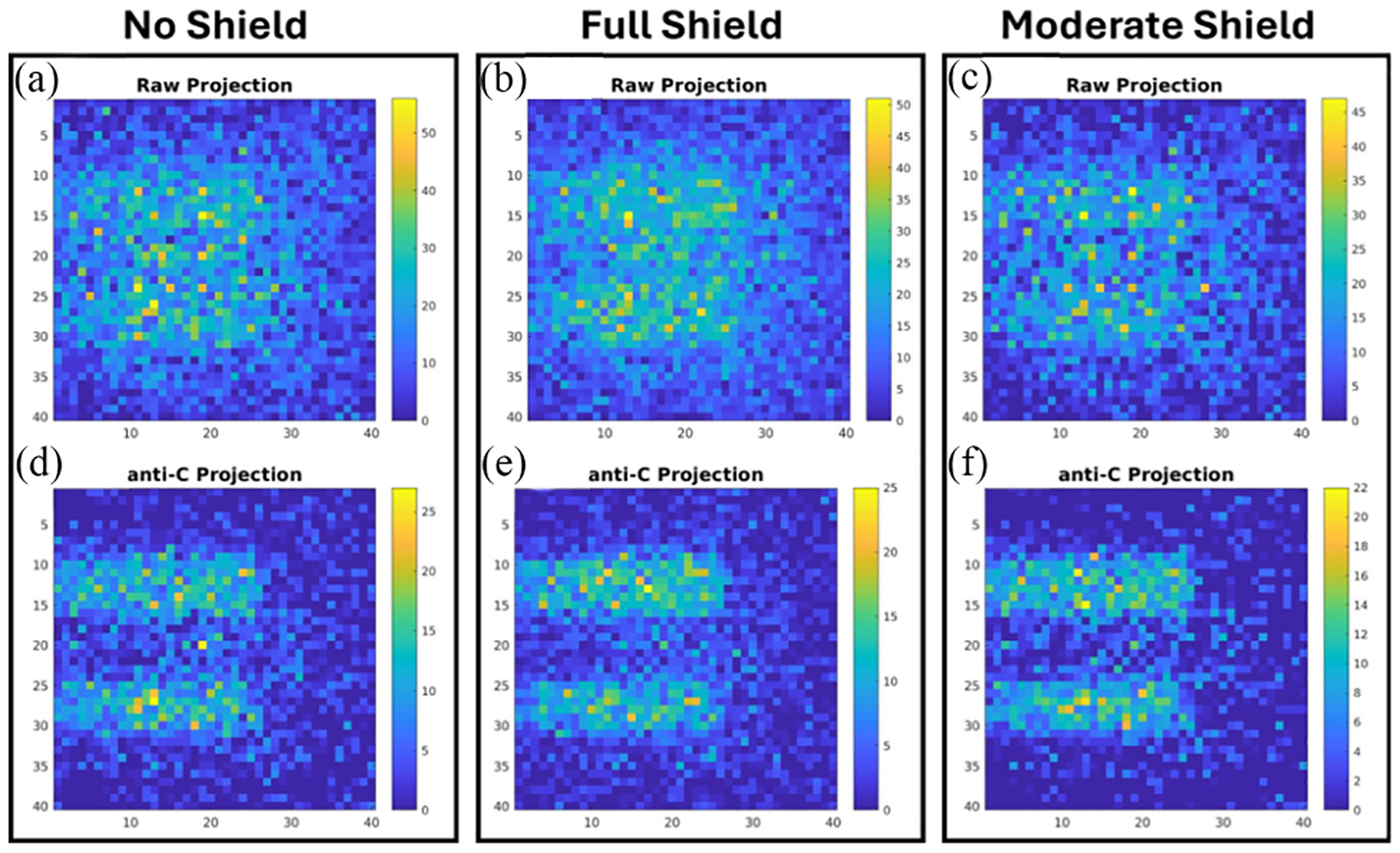
Comparison of raw and anti-coincidence-corrected detector projections under three active shielding configurations. (a)–(c) Raw projection images for no active shielding, full active shielding, and moderate active shielding designs, respectively. (d)–(f) Corresponding anti-coincidence-corrected projection images.

Sing designs are shown in Fig. 8(a)–(f). The anti-coincidence technique significantly suppressed contamination originating from the PET radionuclide in all configurations, revealing the underlying phantom structures with improved clarity.

Quantitative metrics for rejection performance are summarized in Table II. All shielding designs achieved high true rejection fractions (>80%) with only minor differences across configurations. False rejection fractions remained below 19%. The moderate shield design yielded the highest SNR_raw_ (0.29) and NECR_raw_ (41.0 cps), primarily because only the first 6-mm thickness of the SPECT detectors were used for imaging in this design. Compared to 140-keV gamma rays from Tc-99m, 511-keV gamma rays from F-18 penetrate more deeply into the detector. Hence, excluding the 4-mm thickness near the back reduces the contribution of down-scattered events, thereby improving raw noise properties. When the same logic (excluding the back 4-mm layer) is applied retrospectively to the no-shield and full-shield configurations, their SNR_raw_ values improve from 0.29 to 1.38 and 0.24 to 1.47, respectively, while NECR_raw_ increases from 41.0 to 87.0 cps and from 40.9 to 87.6 cps, respectively. The lower SNR_raw_ and NECR_raw_ observed with full shielding are attributed to the extensive coverage of active shielding, which induces additional backscattering into the SPECT detectors.

These findings demonstrate the effectiveness of the anti-coincidence technique across shielding designs and highlight that moderate active shielding achieves a good balance between scatter rejection performance and system complexity.

### B. Evaluation of the Impact of Coincidence Time Window

**Fig. 9.**
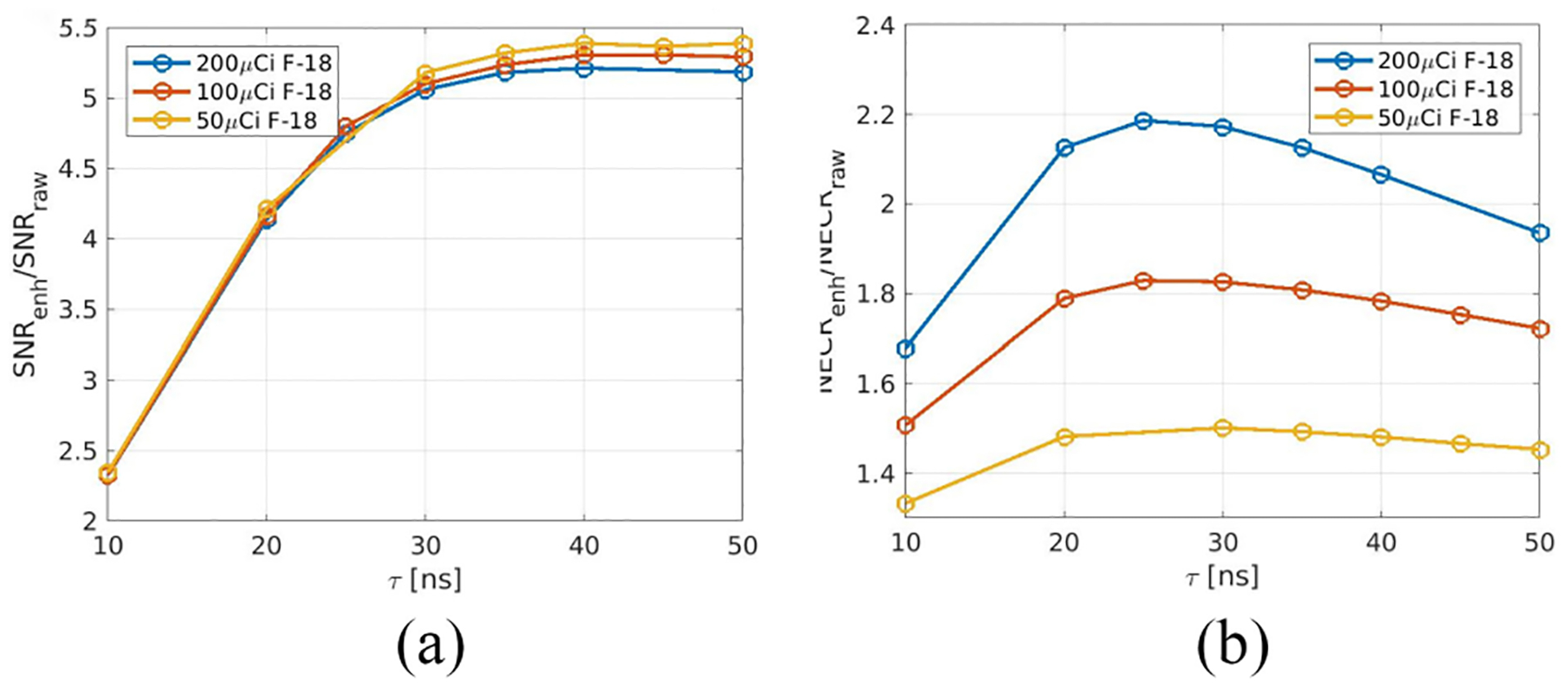
Enhancement of (a) SNR and (b) NECR as a function of the coincidence time window ([− *τ, τ*]) for three PET tracer activities (200, 100, and 50 μCi of F-18).

We investigated how the coincidence time window ([−*τ, τ*]) influences the performance of the anti-coincidence technique across different PET tracer activities. Fig. 9(a) shows the enhancement of SNR (SNR_enh_*/*SNR_raw_) as a function of *τ*. A wider coincidence window increases the likelihood of detecting contamination events in coincidence, resulting in greater scatter rejection and improved SNR. However, this also increases the probability of falsely rejecting true SPECT events due to random coincidences. Fig. 9(b) illustrates the corresponding enhancement of NECR (NECR_enh_*/*NECR_raw_). For each PET tracer activity, there is an optimal *τ*, between 25–30 ns, which maximizes NECR by balancing true rejection and false rejection rates. Higher PET activities (200 μCi) require narrower coincidence windows to minimize random coincidence effects, while lower PET activities (50 μCi) allow for broader windows due to reduced random coincidence rates. It should be noted that, for a given source activity, the SNR_raw_ and NECR_raw_ remain constant across all coincidence time windows, so maximizing the enhancement is mathematically equivalent to maximizing the absolute SNR and NECR values. Therefore, the *τ* value corresponding to the peak enhancement represents the optimal coincidence time window for system performance.

The raw SNR and NECR values for the three PET activities were summarized in Table III. As expected, both SNR_raw_ and NECR_raw_ increased with decreasing PET activity due to reduced down-scatter contamination.

### C. Data Qualities With Different Scatter Correction Methods and Detector Materials

**Fig. 10.**
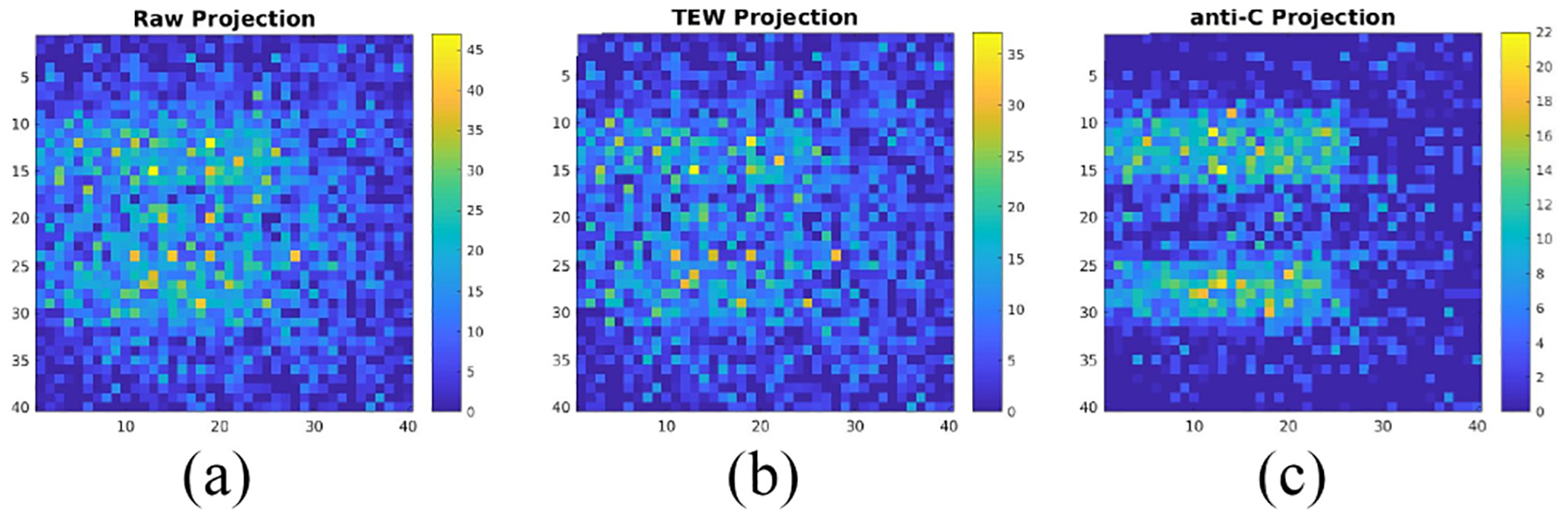
Comparison of projections on a CZT SPECT detector. (a) Raw projection. (b) TEW-corrected projection. (c) Anti-coincidence-corrected projection.

We evaluated the performance of anti-coincidence technique and TEW scatter correction on two SPECT detector materials, such as CZT and GAGG. Representative detector projections for CZT detectors are shown in Fig. 10. The raw projection [Fig. 10(a)] shows heavy down-scatter contamination from F-18. The TEW correction [Fig. 10(b)] reduces contamination but increases statistical noise. In contrast, anti-coincidence [Fig. 10(c)] achieves better scatter suppression and clearer phantom delineation. Although anti-coincidence and TEW are compatible, TEW was not applied to datasets acquired with anti-coincidence in this study because the residual scatter after anti-coincidence rejection was minimal. Furthermore, applying TEW in these low-activity regimes would likely offer limited additional benefits and could amplify statistical noise.

The corresponding results for GAGG detectors are presented in Fig. 12. Compared to CZT, GAGG detectors exhibited higher raw noise levels [Fig. 10(a)] due to their lower energy resolution (10% versus 2% for CZT). TEW [Fig. 12(b)] partially reduces scatter but is limited by spectral broadening. Anti-coincidence [Fig. 12(c)] improves image quality but is hindered by GAGG’s poor energy resolution, admitting more scattered events into the SPECT window.

With 200 μCi of F-18, scatter rejection metrics with the anti-coincidence technique are listed in Table IV. Notably, GAGG’s superior timing resolution (1 ns) and narrower coincidence time window ([−2 *ns*, 2 *ns*]) resulted in a much lower false rejection fraction (3%) compared to CZT (17.9%), indicating its advantage in rejecting random coincidences. However, GAGG exhibits substantially lower SNR and NECR compared to CZT detectors, due to its poorer energy resolution and broader energy window, admitting more scattered events.

### D. Reconstruction With Different Scatter Correction Methods and Detector Materials

**Fig. 11.**
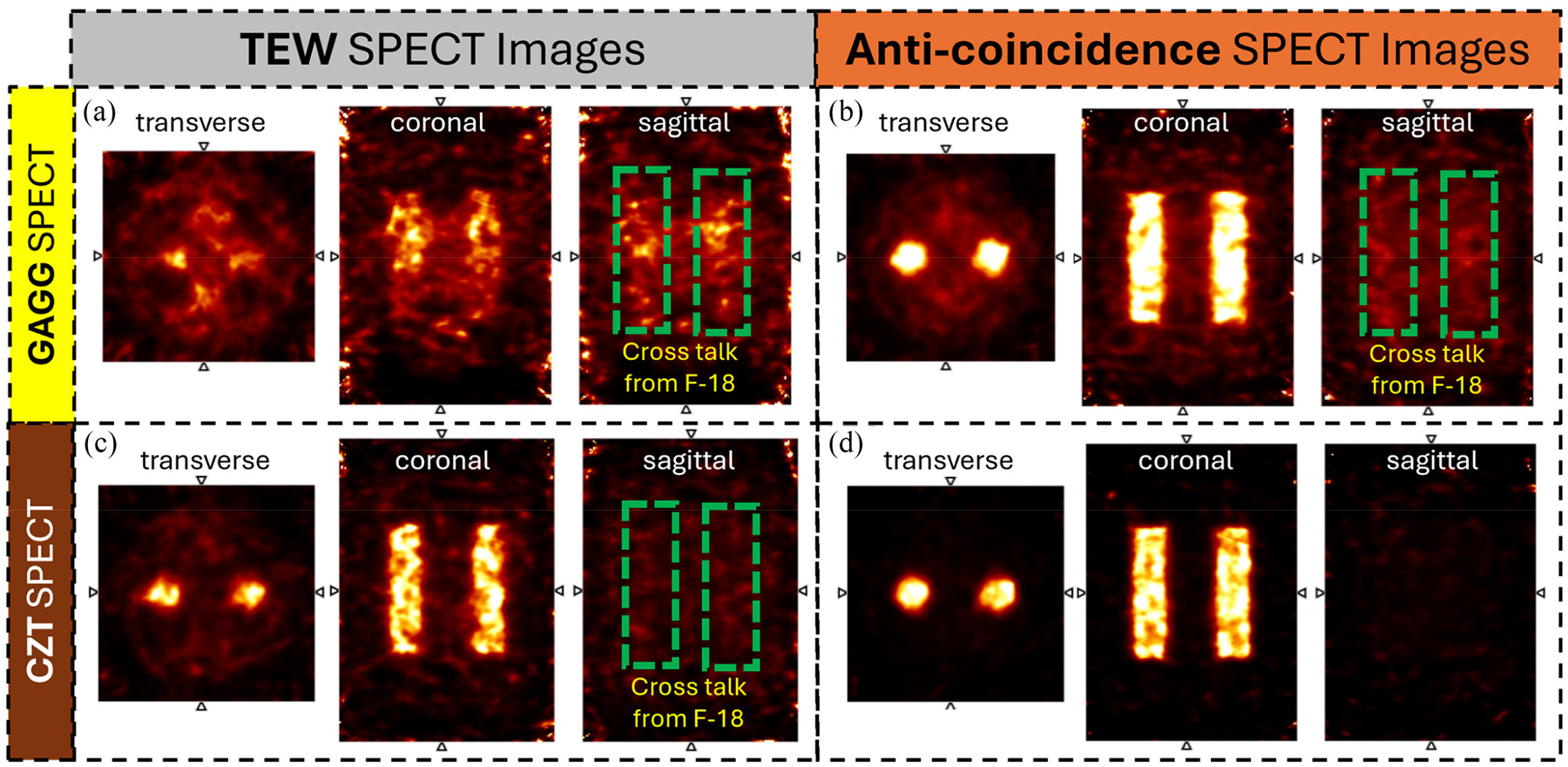
Comparison of reconstructed images: (a) TEW-corrected image with GAGG SPECT, (b) anti-coincidence-corrected image with GAGG SPECT, (c) TEW-corrected image with CZT SPECT, (d) anti-coincidence-corrected image with CZT SPECT. All images are the 15*th* iteration in MLEM. The object space is 128 × 128 × 180 with the voxel size of 0.5 × 0.5 × 0.5 *mm*^3^.

**Fig. 12.**
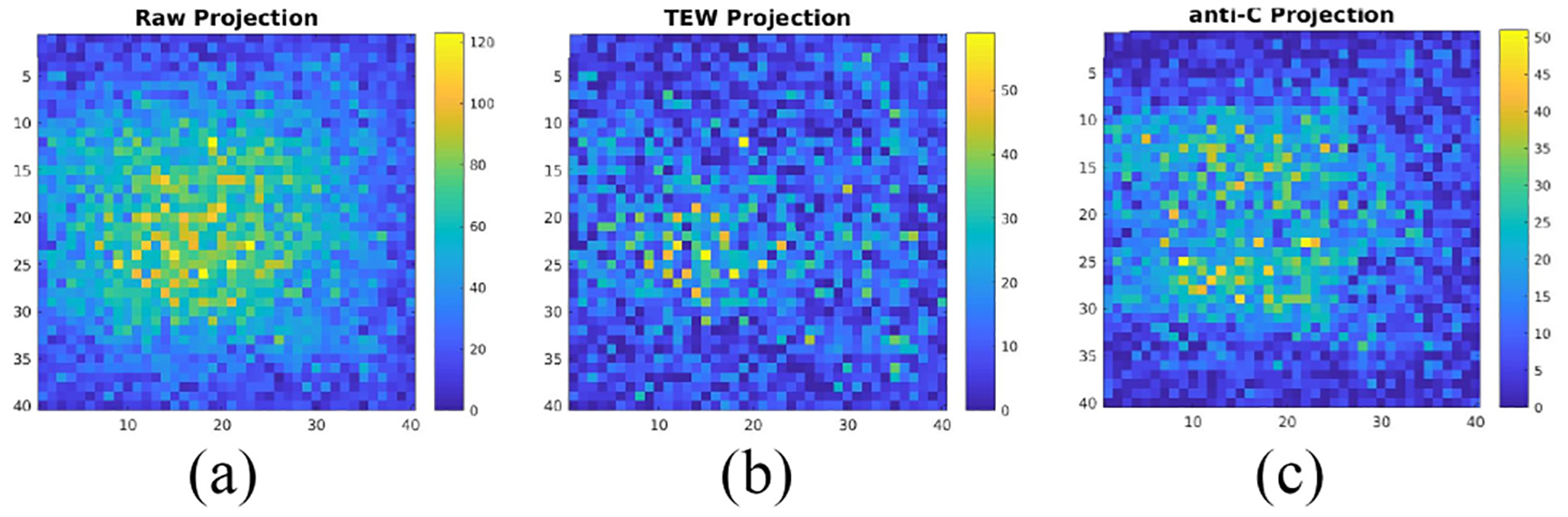
Comparison of projections on a GAGG SPECT detector: (a) raw projection, (b) TEW-corrected projection, and (c) anticoincidence-corrected projection.

We evaluated the impact of scatter correction methods (TEW versus anti-coincidence) and SPECT detector materials (CZT versus GAGG) on reconstructed images. As shown in Fig. 11, TEW-corrected images and the anti-coincidence-corrected image with GAGG exhibit residual crosstalk from F-18 PET tracers (highlighted in green dashed boxes). In contrast, the anti-coincidence-corrected image with CZT [Fig. 11(d)] shows effective reduction of scatter contamination.

**Fig. 13.**
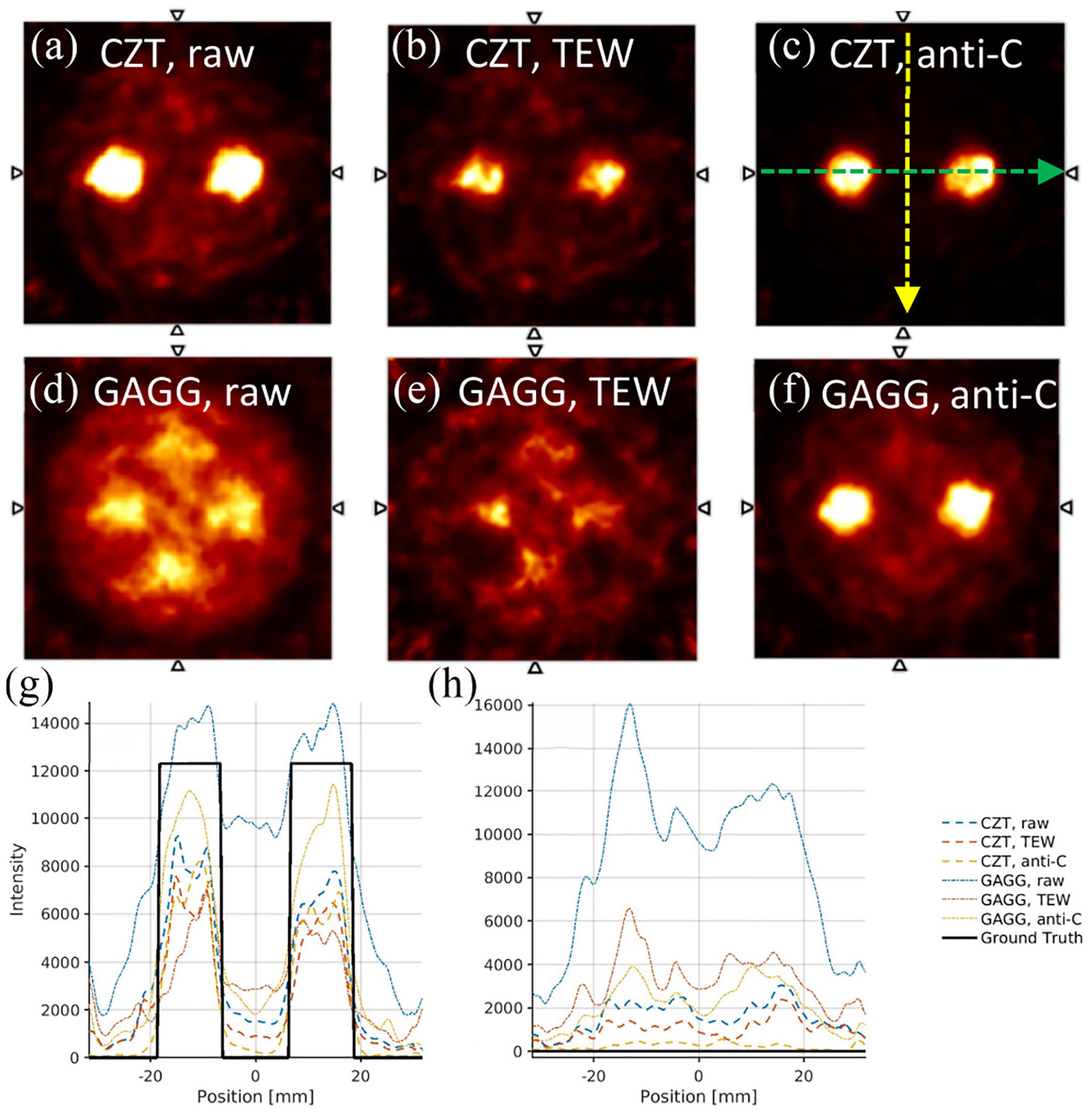
Comparison of reconstructed SPECT images and line profiles. (a)–(c) CZT images: raw, TEW-corrected, and anti-coincidence-corrected. (d)–(f) GAGG images: raw, TEW-corrected, and anti-coincidence-corrected. (g) and (h) Intensity profiles along the green (horizontal) and yellow (vertical) dashed arrows in (c). The ground truth profile is overlaid in black for reference.

Fig. 13 further highlights these differences through line profiles extracted along horizontal (green) and vertical (yellow) dashed lines in Fig. 13(c). The raw profiles show elevated background due to scatter contamination. The TEW correction reduces this background but fails to fully recover contrast between the hot rods and surrounding areas. In contrast, anti-coincidence correction recovers the expected profile shape with reduced background and improved correspondence to the ground truth.

Quantitative metrics in Table V confirm these trends. For CZT, the contrast increased from 0.84 (TEW) to 0.96 (anti-coincidence), while GAGG achieved a smaller improvement (0.56 to 0.77). The anti-coincidence image with CZT achieves the lowest noise level, demonstrating superior noise suppression and overall performance.

## IV. Discussion

In this study, we proposed and evaluated an anti-coincidence scatter rejection technique combined with active shielding for simultaneous PET/SPECT imaging, addressing the persistent challenge of down-scatter contamination from PET radiotracers in SPECT images. Simulation studies were based on a preclinical PET/SPECT system design that efficiently utilizes the gaps between PET detectors to accommodate SPECT cameras, ensuring sufficient angular sampling and sensitivity for both modalities. The current system design features transverse FOVs of 8 cm for PET and 6 cm for SPECT, with PET for whole-target coverage, and SPECT dedicated to high-resolution imaging within a smaller, targeted region. Although the hybrid configuration introduces a modest PET sensitivity reduction from ~16.8% to ~14% due to the SPECT inserts, due to its long axial FOV, its overall sensitivity remains slightly higher than most preclinical PET. The estimated PET spatial resolution is comparable to most standalone systems [57], [58]. Similarly, the current SPECT subsystem achieves sensitivity and spatial resolution consistent with those reported for preclinical SPECT systems [59], [60], [61].

Because the primary objective of this study is to demonstrate the anti-coincidence acquisition technique, rather than to optimize hardware performance, we focus on quantitative evaluation metrics and omit phantom images of spatial resolution and sensitivity for brevity. The current configuration serves as an early-stage design that validates the feasibility of simultaneous PET/SPECT imaging and highlights the tradeoffs among shielding configurations, coincidence time windows, and detector materials. These results provide valuable guidance for the future development of integrated hybrid systems. Further optimization could expand the SPECT coverage by adjusting the pinhole geometry—for example, increasing the open angle or introducing pinhole tilt to enlarge the transverse FOV. A comprehensive system characterization will be presented in a dedicated performance-evaluation study.

The results demonstrate that the anti-coincidence technique effectively suppresses scatter contamination, particularly in low uptake SPECT imaging scenarios where traditional methods, such as TEW fail. Anti-coincidence operates at the acquisition level, allowing for real-time rejection of scatter events without relying on high-count statistics or computationally intensive post-processing.

Active shielding enhances anti-coincidence performance by increasing the likelihood of detecting scattered highenergy photons that would otherwise escape from the system. However, its benefit can be partially compensated by extensive PET detector coverage. Our results show that even without active shielding, anti-coincidence alone achieves an SNR of 1.38 and a NECR of 87.0 cps, indicating that a plain PET/SPECT system can still benefit substantially from this method. This is especially advantageous for system designs where adding active shielding may be impractical due to space, cost, or cooling constraints. For systems aiming for optimal performance, a moderate active shielding design provides a good balance between scatter rejection efficiency and implementation complexity.

The enhancement of SNR and NECR with varying coincidence time windows revealed important tradeoffs between true event retention and false rejection (Fig. 9). For PET tracers with high activities, narrower coincidence windows are required to limit random coincidences, whereas lower activity levels allow for wider windows to maximize true rejection. The mathematical framework provided in Section II-D can serve as a guideline for optimizing these parameters in practical system implementations.

The choice of SPECT detector material critically influences the performance of anti-coincidence rejection. CZT detectors, with superior energy resolution (2%), enable precise spectral discrimination and reduced contamination in the SPECT primary energy window, resulting in higher SNR and NECR after correction. However, their relatively poor timing resolution (25 ns) increases susceptibility to random coincidences, leading to higher false rejection fractions. In contrast, GAGG detectors offer fast timing (1 ns), which reduces random rejection, but their poor energy resolution (10%) admits more scattered photons, limiting the effectiveness of anti-coincidence. These results highlight a fundamental tradeoff between timing and energy resolution in the preclinical context of simultaneous PET/SPECT imaging and informs the optimal detector selection for systems prioritizing contamination rejection.

Compared to TEW, anti-coincidence consistently provided superior suppression of PET crosstalk and improved contrast in both projection and reconstructed images. TEW suffers from increased statistical noise and poor performance in low-count conditions, especially with isotopes, such as Ac-225 or Lu-177. By contrast, anti-coincidence is particularly effective in low-activity regimes. Moreover, a key strength of the anti-coincidence approach is that it rejects scatter events intrinsically during acquisition, making it fully compatible with post-processing scatter correction methods, such as TEW or model-based corrections, enabling layered correction strategies for challenging multi-isotope imaging scenarios. In this study, we did not apply TEW following anti-coincidence because the residual scatter after anti-coincidence rejection is already minimal, and TEW’s reliance on auxiliary energy windows would likely amplify statistical noise rather than improve image quality under these conditions. However, in high-uptake scenarios where anti-coincidence performance may be limited by random coincidences, combining anti-coincidence with post-processing scatter correction methods like TEW could offer complementary benefits.

This study was based on simulation results and focused on idealized system geometries and phantom models. While the current system design targets preclinical imaging, the proposed anti-coincidence strategy and shielding optimization principles are directly applicable to clinical PET/SPECT systems. Practical implementation will require consideration of hardware constraints, such as detector timing jitter, shielding tradeoffs, and acquisition system capabilities.

The proposed hybrid PET/SPECT system was simulated based on existing detector technologies that are already feasible for experimental implementation. The PET subsystem employs pixelated BGO detectors, which are well established and provide high stopping power and stable timing performance [51]. The SPECT subsystem utilizes DOI-capable CZT detectors, which offer excellent energy resolution but currently have limited timing performance in standard implementations [52], [53], [54]. However, we have demonstrated that CZT detectors can achieve timing resolutions on the order of 10 ns under optimized electronic configurations, indicating that the required coincidence timing is technically achievable [62]. The next step would be to develop and integrate synchronized readout electronics for BGO and CZT detectors to support consistent coincidence timing across modalities.

In practice, additional nonideal factors, such as electronic noise, temperature drift, and mechanical misalignment, may cause deviations from simulation performance by affecting spatial resolution or introducing minor artifacts. These effects, however, are common to all imaging systems and can be mitigated through precise calibration and robust system modeling. Future work will focus on constructing a prototype hybrid PET/SPECT system, validating anti-coincidence performance under realistic multitracer distributions, and optimizing coincidence logic for real-time scatter rejection. The integration of anti-coincidence with advanced post-processing scatter correction algorithms will also be explored to further enhance image quality in high-count and complex clinical imaging scenarios.

## V. Conclusion

This study proposed and evaluated the anti-coincidence scatter rejection technique, combined with active shielding, to suppress down-scatter contamination in simultaneous PET/SPECT imaging. Using GEANT4 simulations, we demonstrated that anti-coincidence effectively improves SPECT image quality by rejecting contamination events during acquisition. In a preclinical PET/SPECT system design, anti-coincidence enhanced SNR from 0.29 to 1.48 and NECR from 41.3 to 89.1 cps with moderate active shielding. Even without active shielding, anti-coincidence alone achieved substantial improvements, highlighting its practicality for systems where shielding implementation is constrained.

Compared to traditional methods reliant on high counting statistics, anti-coincidence achieved superior scatter suppression and proved especially effective in low-uptake scenarios, which are critical for quantitative imaging of therapeutic tracers and dosimetry applications. Notably, as an acquisition-level technique, anti-coincidence is also compatible with post-processing scatter correction methods, offering a flexible solution for future hybrid PET/SPECT systems. Future work will focus on constructing a prototype hybrid PET/SPECT system and experimentally validating the proposed anti-coincidence technique, as well as extending the design principles to clinical systems for multitracer and theragnostic imaging.

## Figures and Tables

**Fig. 7.**
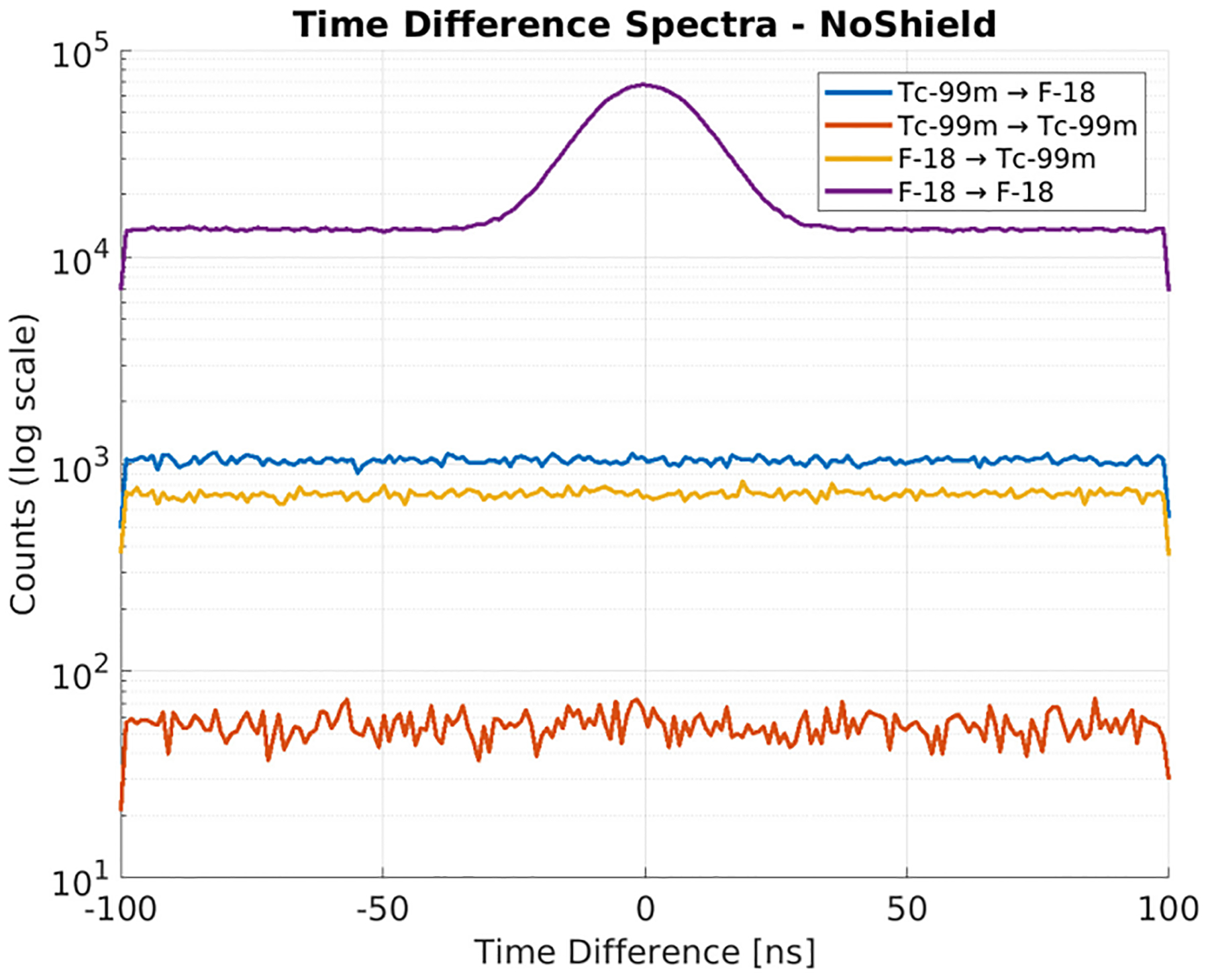
Time-difference spectra with the no-shield setup from 20 μCi Tc-99m and 200 μCi F-18 and 1200-s acquisition. Based on the sources of primary and secondary events, four primary → secondary categories of spectra are shown: Tc-99m → F-18, Tc-99m → Tc-99m, F-18 → Tc-99m, and F-18 → F-18.

**TABLE I:** Simulation Parameters for PET and SPECT Detectors Used in the Simultaneous PET/SPECT System

Modality/Detector	PET/BGO	SPECT/CZT
Energy Resolution	20% FWHM	2% FWHM
Size of BGO/CZT element	0.9 × 0.9 × 20 mm^3^	0.5 × 0.5 × 1 mm^3^
Timing Resolution	1 ns FWHM	25 ns FWHM
Coincidence Time Window	[-30 ns, 30 ns]	

**TABLE II:** Quantitative Metrics of Scatter Rejection for Three Active Shielding Configurations

Metrics	False rej.	True rej.	*SNR*_*raw*_	*SNR*_*enh*_	*NECR*_*raw*_	*NECR*_*enh*_
**No Shield**	17.0%	80.4%	0.21	0.94	33.9 cps	78.4 cps
**Full**	18.6%	84.0%	0.21	1.00	33.7 cps	79.4 cps
**Moderate**	17.9%	83.7%	0.29	1.48	41.3 cps	89.1 cps

**TABLE III:** Raw SNR and NECR values for Three PET Tracer Activities

F-18 Activity	200 μCi	100 μCi	50 μCi
***SNR***_***raw***_	0.29	0.58	1.17
***NECR***_***raw***_	41.0 cps	66.9 cps	97.6 cps

**TABLE IV:** Quantitative Metrics for Scatter Rejection Using CZT and GAGG SPECT Detectors With the anti-Coincidence Technique

Metrics	False rej.	True rej.	*SNR*_*raw*_	*SNR*_*enh*_	*NECR*_*raw*_	*NECR*_*enh*_
**CZT**	17.9%	83.7%	0.29	1.48	41.3 cps	89.1 cps
**GAGG**	3%	75.0%	0.073	0.28	15.9 cps	50.0 cps

**TABLE V:** Quantitative Metrics for Reconstructed SPECT Images With GAGG and CZT Detectors Using TEW and Anti-Coincidence Scatter Correction Methods

Detector Material	CZT			GAGG		
Dataset	Raw	TEW	anti-coincidence	Raw	TEW	anti-coincidence
Contrast	0.78	0.84	0.96	0.36	0.56	0.77
Noise Level	1508	855	257	7956	1860	2064
